# Efficient Neuroprotective Rescue of Sacsin-Related Disease Phenotypes in Zebrafish

**DOI:** 10.3390/ijms22168401

**Published:** 2021-08-05

**Authors:** Valentina Naef, Maria Marchese, Asahi Ogi, Gianluca Fichi, Daniele Galatolo, Rosario Licitra, Stefano Doccini, Tiziano Verri, Francesco Argenton, Federica Morani, Filippo M. Santorelli

**Affiliations:** 1 Neurobiology and Molecular Medicine, IRCCS Fondazione Stella Maris, 56128 Pisa, Italy; asahi.ogi@vet.unipi.it (A.O.); daniele.galatolo1408@gmail.com (D.G.); rosario.licitra@vet.unipi.it (R.L.); stefanodoccini@gmail.com (S.D.); 2 Struttura Complessa Toscana Sud (Grosseto), Istituto Zooprofilattico Sperimentale del Lazio e Toscana M. Aleandri, 58100 Grosseto, Italy; gianluca.fichi@gmail.com; 3 Department of Biological and Environmental Sciences and Technologies, University of Salento, 73100 Lecce, Italy; tiziano.verri@unisalento.it; 4 Department of Biology, University of Padua, 35131 Padua, Italy; francesco.argenton@unipd.it; 5 Department of Biology, University of Pisa, 56124 Pisa, Italy; morani.federica@gmail.com

**Keywords:** ARSACS, ataxia, cerebellum, neurological disorders, zebrafish

## Abstract

Autosomal recessive spastic ataxia of Charlevoix-Saguenay (ARSACS) is a multisystem hereditary ataxia associated with mutations in *SACS*, which encodes sacsin, a protein of still only partially understood function. Although mouse models of ARSACS mimic largely the disease progression seen in humans, their use in the validation of effective therapies has not yet been proposed. Recently, the teleost *Danio rerio* has attracted increasing attention as a vertebrate model that allows rapid and economical screening, of candidate molecules, and thus combines the advantages of whole-organism phenotypic assays and in vitro high-throughput screening assays. Through CRISPR/Cas9-based mutagenesis, we generated and characterized a zebrafish *sacs*-null mutant line that replicates the main features of ARSACS. The *sacs*-null fish showed motor impairment, hindbrain atrophy, mitochondrial dysfunction, and reactive oxygen species accumulation. As proof of principle for using these mutant fish in high-throughput screening studies, we showed that both acetyl-DL-leucine and tauroursodeoxycholic acid improved locomotor and biochemical phenotypes in *sacs*^−/−^ larvae treated with these neuroprotective agents, by mediating significant rescue of the molecular functions altered by sacsin loss. Taken together, the evidence here reported shows the zebrafish to be a valuable model organism for the identification of novel molecular mechanisms and for efficient and rapid in vivo optimization and screening of potential therapeutic compounds. These findings may pave the way for new interventions targeting the earliest phases of Purkinje cell degeneration in ARSACS.

## 1. Introduction

Autosomal recessive spastic ataxia of Charlevoix-Saguenay (ARSACS) is a rare early-onset neurodegenerative disease associated with mutations in the *SACS* gene, which encodes sacsin, a 520 kDa multidomain protein [1]. Affected patients present spasticity, paraparesis, early onset ataxia, and distal muscle wasting. Moreover, both previous [2] and more recent [3] studies show that retinal fiber hypermyelination could be considered an early marker of the disease [2]. Over 200 mutations in *SACS* have been identified, most leading to complete loss of sacsin function [3]. Sacsin is one of the largest proteins encoded by the human genome [4], and to date its multidomain secondary structure has been revealed only in part. From the N- to the C-terminal, sacsin is reported to be composed of a ubiquitin-like domain that binds to the proteasome, three large sacsin repeats forming a “sacsin repeating region” (SRR) that may have an Hsp90-like chaperone function [5], a xeroderma pigmentosum complementation group C binding (XPCB) domain that binds to the Ube3A ubiquitin protein ligase, a DnaJ domain binding Hsc70, and a higher eukaryotes and prokaryotes nucleotide-binding (HEPN) domain mediating sacsin dimerization and binding to nucleotides or their analogs [1,5,6]. Notably, the SRR has recently been found to be part of a larger sacsin internal repeat (SIRPT) region [7]. The nature of these modules and their architecture suggest that sacsin may be involved in protein quality control, and thus play a role in neurodevelopment and neurodegeneration. Despite the identification of the above-mentioned regions, the functional role of *SACS* and the pathophysiological consequences of its dysfunction remain largely uncharacterized. Using cell lines (SH-SY5Y, He-La, Cos-7), patient skin fibroblasts, primary neuron cultures, knock-out (KO) mouse models, and organotypic murine brain slice cultures, it has been shown that sacsin is expressed on the mitochondrial surface [8]. Of note, sacsin deficiency in cultured skin fibroblasts from ARSACS patients leads to alterations in mitochondrial morphology and function [8], while sacsin knockdown in primary hippocampal cultures causes clustering of mitochondria, which accumulate in the soma and proximal dendrites [8]. In addition, converging evidence from multiple studies points to sacsin involvement both in regulating mitochondrial dynamics, and in the organization of intermediate filaments, functions that are closely related, as indicated by the importance of mitochondrial network and function regulation at the cytoskeletal level [9,10]. Deep phenotyping and neuropathological studies in *Sacs*^−/−^ transgenic mice have shown pathological changes in the synaptic compartment, electric impairment of Purkinje cells (PCs) with significant neuronal cell loss in the cerebellum, as well as motor deficits reminiscent of ataxia, all features that replicate those seen in ARSACS patients [4,8,10,11]. By means of transcriptomic analysis, we previously confirmed the presence of mitochondrial dysfunction associated with increased oxidative stress in sacsin-depleted cells, providing the first demonstration of an autophagic pathway impairment in these cells and suggesting that impaired autophagic flux could be the element linking the chaperone-like function of sacsin with its role in mitochondrial dynamics [12]. Even more recently, using an original proteomic approach in neuronal-like cells, we identified significant dysregulation of biological processes related to neuroinflammation, synaptogenesis, and engulfment of cells in ARSACS, these findings further reinforcing the hypothesis of a role for sacsin in neurodevelopment [13]. ARSACS remains an incurable disorder and there is an urgent need to define new therapies. In this context, high-capacity in vivo screening of candidate drugs/compounds would be useful both for optimizing compounds and for prioritizing their subsequent testing in mammalian models. The teleost *Danio rerio* (zebrafish) has recently emerged as an attractive platform in preclinical research of neurodevelopmental and neurodegenerative disorders [14,15,16,17] not least because it complies with the 3R principles of animal research [18,19,20]. The zebrafish is poised to be an important model in bridging the gap between in vitro assays and in vivo studies in mammals [21,22]. It combines invertebrate-like genetics with vertebrate brain structures, and its transparency during embryonic development has been exploited in order to directly unveil, in vivo, the crucial structure–physiology–function relationships in the vertebrate brain [23,24]. In addition, its high fecundity and rapid development are also significant advantages, allowing rapid in vivo exploration of potential therapeutic drugs [25]. Therefore, with the dual aim of increasing our firepower in the battle against ARSACS and better characterizing biochemical alterations driven by the absence of sacsin, we developed a new loss-of-function vertebrate model in order to shed further light on the role of sacsin in early neurodevelopment, and evaluate the efficacy of the system in drug screening. Using the CRISPR/Cas9 technology [26], we generated a zebrafish *sacs*^−/−^ mutant line and observed that the model displayed motor impairment, hindbrain atrophy, mitochondrial dysfunction, and oxidative stress, mimicking features already seen in human cells and mouse models. We suggest that our *sacs^−/−^* KO strain may prove useful for the translation of potential therapies to ARSACS patients, as it decreased the need for time-consuming and labor-intensive procedures.

## 2. Results

### 2.1. Generation of Sacs-Null Mutant Zebrafish

**Figure 1:**
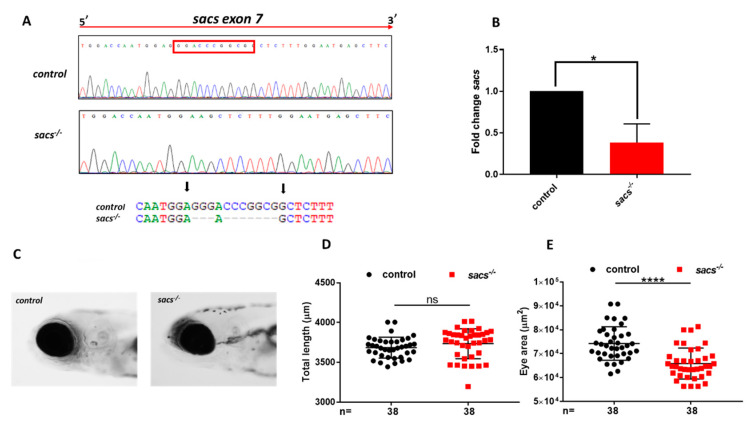
Generation of sacs-null mutant zebrafish. (**A**) Sequencing chromatographs of control and sacs-null mutant (*sacs*^−/−^)specimens and alleles. The red rectangle define frameshift mutation in exon 7. The arrow indicates the area of the induced deletion. (**B**) qRT-PCR analysis revealed a decrease in the level of sacs mRNA expression, normalized to β-actin. Three independent RNA samples from *sacs*^−/−^ mutant larvae at 120 hpf and from controls were analyzed. * *p* ≤ 0.05, calculated by Student’s *t*-test. (**C**) Lateral view photographs of representative control and *sacs*^−/−^ specimens. (**D**) No dysmorphology and full length was noted at 120 hpf, but (**E**) homozygous larvae showed slight but significant “microphthalmia”, **** *p* ≤ 0.00001, calculated by Mann-Whitney test. The values are expressed as mean ± standard deviation (SD). Abbreviations: *n*, total number of evaluated embryos; error bars indicate standard errors of the means; ns, not significant.

The zebrafish sacs gene (ENSDARG00000091042.4), which maps to chromosome 15, consists of 9 coding exons, and encodes a 4578 amino acid molecular chaperone protein. Previously, a bioinformatic analysis revealed that sacsin is conserved in vertebrates [7]. Notably, however, one homologous sequence of sacs can be found in zebrafish on chromosome 9 (predictive si:dkeyp-118h9.7: transcript ID ENSDART00000162761.2). This variant, which, for simplicity, we will call sacs2, is probably the result of a whole genome duplication event that occurred during teleost fish evolution, and it implies that in zebrafish, the specific functions of sacsin could be divided between the two duplicated genes, or simply lost or disrupted for one of them. Performing an in-silico analysis of the amino acid sequence of sacsin in *Danio rerio* (both sacs and sacs2), *Homo sapiens*, and *Mus musculus*, we observed high conservation of the human protein in the zebrafish sacs (70% conservation, 35% identity) but not in the sacs2 form (32% conservation, 24% identity) (Supplementary Materials Figure S1), which suggests that sacs is actually the fish ortholog of the human protein. Thus, by means of CRISPR/Cas9 editing, we engineered sacs-null mutants that generated a 10-bp deletion mutation in exon 7 of the sacs gene leading to a frameshift mutation and premature stop codon at residue 495 (R487Kfs*495) (Figure 1A), expected to disrupt the structure of sacsin near the N-terminus. The homozygous sacs^−/−^ F2 zebrafish mutant line was raised to adulthood and its progeny (F3) were used to investigate the phenotype of the mutant strain and determine whether it replicates the features of ARSACS. Due to the lack of a reliable antibody for zebrafish, it was not possible to test the protein abundance in the mutant zebrafish by Western blotting. However, qRT-PCR analysis showed a notable decrease in the amount of sacs transcript [27] in mutants compared with control (wild-type) siblings at 120 hpf (Figure 1B). The homozygous sacs mutant zebrafish larvae at 120 hpf showed slight “microphthalmia” (Figure 1C–E).

### 2.2. Sacs^−/−^ Mutant Zebrafish Displays Motor Impairment and Reduced “Cerebellar” Area

**Figure 2:**
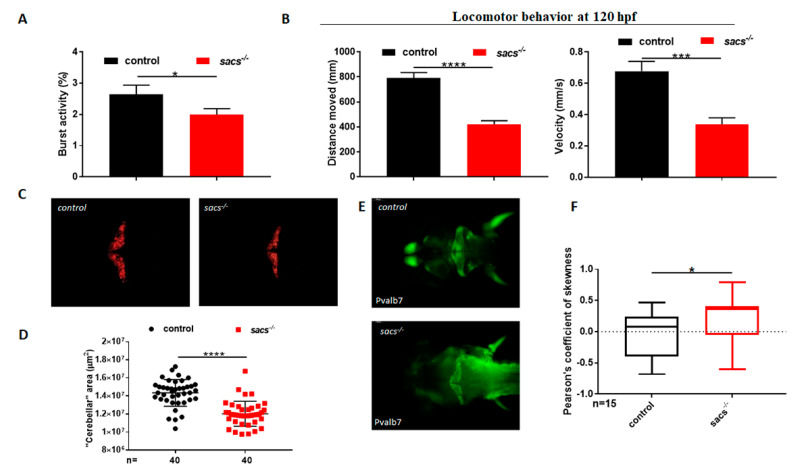
*sacs^−/−^* mutant zebrafish display motor impairment and reduced cerebellar area. (**A**) Coiling frequency in zebrafish embryos at 30 hpf is decreased in *sacs^−/−^* compared with control specimens (*sacs^−/−^ n* = 113; controls *n* = 113, in 4 independent experiments). (**B**) Automated analysis of spontaneous motor activity revealed a reduction in swim distance and velocity in *sacs^−/−^* larvae at 120 hpf compared with observations in control siblings (*sacs^−/−^ n* = 256; controls *n* = 273, in 5 independent experiments). Statistical analysis (* *p* ≤ 0.05, *** *p* ≤ 0.001, **** *p* ≤ 0.0001) was performed using the Mann-Whitney test. (**C**) “Cerebellar” morphology as assessed in vivo by RFP fluorescence in Tg(tagRFP-T:PC:GCaMP5G). The “cerebellar” area was found to be significantly reduced in *sacs^−/−^* compared with control specimens at 120 hpf. (**D**) Statistical analysis of the data shown in (**C**), **** *p* ≤ 0.00001, calculated by the Mann-Whitney test. The values are expressed as mean ± standard deviation. (**E**) Dorsal views at 120 hpf of whole-mount larvae labeled with mAB pvalb7 (*sacs^−/−^ n* = 20; controls *n* = 20). (**F**) Pearson’s coefficient of skewness of the distribution of the fluorescence fluctuations, ∆F/F0. These statistics are cumulated from 16 recordings in controls and *sacs^−/−^* mutants, respectively. (* *p* ≤ 0.05 was calculated using the Mann-Whitney test). Abbreviations: *n*, number of evaluated embryos in total; error bars indicate standard errors of the means.

Impaired motor coordination is a clinical hallmark of ARSACS in children. The transgenic *Sacs*^−/−^ mouse model displays ataxia-like motor impairment as well as PC death [10]. We analyzed the locomotor behavior of *sacs*-null (*sacs*^−/−^) mutant zebrafish larvae. Zebrafish embryos develop rapidly and show their first spontaneous movement (slow, alternating tail flicks) at approximately 17 hpf [28]. Analysis of tail flicks at 30 hpf in *sacs^−/−^* embryos showed a significant decrease in burst activity (i.e., the percentage of time an embryo is moving) compared with controls (Figure 2A). At 120 hpf, video tracking data revealed significantly reduced locomotor activity of *sacs*^−/−^ larvae compared with control siblings, with the mutant larvae showing less movement in terms of both velocity and distance covered (Figure 2B, Supplementary Materials Video S1). To explore the nature of this motor deficit, we investigated the morphology of spinal motor neurons using the motor axon marker syt2. Immunolabeling of spinal motor neuron axons in 48 hpf mutant and control embryos showed no alterations in axon outgrowth or arborization, and no reduction of axon length (Supplementary Materials Figure S1). Acetylated α-tubulin staining confirmed the absence of motor and sensory neuron abnormalities (Supplementary Materials Figure S1). In addition, birefringence assay showed no muscle structure or compaction abnormality (Supplementary Materials Figure S1). A common feature of ARSACS is the presence of upper cerebellar vermis atrophy evolving into cerebellar hemisphere atrophy as the disease progresses, and it is observed on human brain imaging and post-mortem studies, with accompanying increased PC death [29]. The vertebrate cerebellum is responsible for coordinating body posture, balance, and locomotor control. The cytoarchitecture and connectivity of cerebellar neurons are highly conserved between teleosts and mammals [24], and several studies have shown that the “cerebellum” of zebrafish larvae plays a functional role relating to motor coordination, adaptation, and learning [30]. This larval “cerebellum” lends itself to study of the physiology and function of PCs [24,31,32], which in zebrafish develop fast, first emerging at 56 hpf, and then increasing in number until 7 dpf [33]. To test PCs in *sacs*^−/−^, we created a null line in the genetic background of a stable transgenic strain: Tg(tagRFP-T:PC:GCaMP5G). The latter, due to a PC-specific enhancer element, makes it possible to visualize PCs in vivo [24]. At 120 hpf, *sacs^−/−^* larvae displayed a significant reduction in the “cerebellar” area (Figure 2C,D); immunodetection of the calcium (Ca^2+^) ion-binding protein pvalb7 produced a similar finding (Figure 2E), i.e., suggesting a reduction in the number of PCs. In the cerebellum of adult mice, Pvalb is expressed in PCs and molecular layer interneurons; instead, the zebrafish form, pvalb7, is expressed in PC dendrites, soma, and axons [34], and used to monitor hindbrain and optic tectum development accounting for cerebellar size and morphology.

As observed in other forms of inherited ataxia in mammals, such as murine models of SCA2 [35], impairment of Ca^2+^ signaling can lead to death of PCs [35]. Indeed, intracellular Ca^2+^ is a key regulator of the neuronal life cycle and of Ca^2+^ homeostasis maintenance, and not only supports normal brain physiology but also maintains neuronal integrity and long-term cell survival [36]. Therefore, the use of the aforementioned *sacs^−/−^* larvae raised on the transgenic line Tg(tagRFP-T:PC:GCaMP5G) allowed us also to monitor intracellular Ca^2+^ dynamics in real time, and the distribution of the fluorescence fluctuations (ΔF/F0) of Ca^2+^ [37] through the Ca^2+^indicator (GCaMP5G) [24]. In our model, we observed a significant increase in Ca^2+^ fluorescence fluctuations in the larval PCs, the region likely corresponding to the human upper vermis (Figure 2F).

### 2.3. Sacs^−/−^ Mutant Zebrafish Manifest Mitochondrial and Autophagic Dysfunction and ROS Accumulation

**Figure 3:**
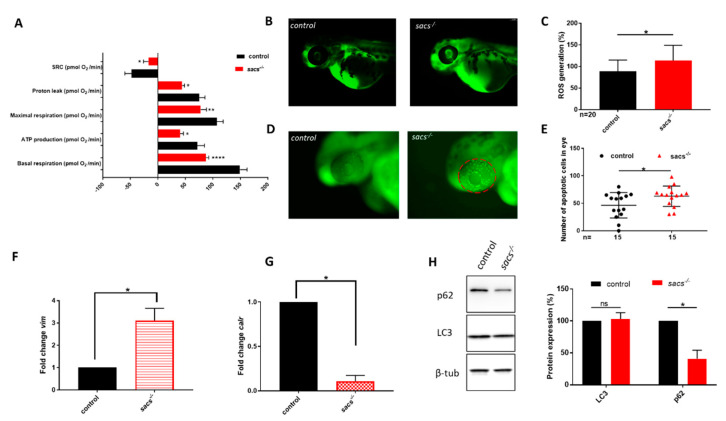
*Sacs^−/−^* mutant zebrafish manifest mitochondrial and autophagic dysfunction and ROS accumulation. (**A**) Mitochondrial respiratory analysis of controls (*n* = 30) and *sacs^−/−^* mutant larvae (*n* = 37) at 120 hpf. * *p* ≤ 0.05, ** *p* ≤ 0.01, **** *p* ≤ 0.0001, calculated by the Mann-Whitney test. (**B**) Representative fluorescence images of ROS generation in zebrafish larvae at 48 hpf. (**C**) Quantitative analysis of ROS generation. * *p* ≤ 0.05 was calculated by the Mann-Whitney test. The values are expressed as mean ± standard deviation (SD). (**D**) Detection of apoptotic cells by acridine orange staining at the level of the eye in controls and *sacs^−/−^* mutant embryos at 48 hpf (lateral views). Apoptotic cells were counted in the area defined by the red circle. (**E**) Quantitative analysis of apoptotic cells. * *p* ≤ 0.05 was calculated by the Mann-Whitney test. The values are expressed as mean ± standard deviation (SD). (**F**,**G**) qRT-PCR analysis revealed increases in *vim* and *calr* expression, once the mRNA expression levels had been normalized to *β-actin*. Three independent RNA samples from controls and *sacs^−/−^* mutant larvae at 120 hpf were analyzed. * *p* ≤ 0.05, calculated by Student’s *t*-test. (**H**) Three independent larval homogenates from controls (*n* = 50) and *sacs^−/−^* larvae (*n* = 50) were tested by Western blotting for the expression of p62 and LC3 proteins. The levels of the different proteins were normalized to β-tubulin. * *p* ≤ 0.05 was calculated by Student’s *t*-test. Abbreviations: *n*, number of evaluated embryos in total; error bars indicate standard error of the mean; ns, not significant; SRC, spare respiratory capacity; ROS, reactive oxygen species.

In previous [38] and more recent [12] experiments in ARSACS, we and others confirmed the presence of decreased mitochondrial function associated with increased oxidative stress, and also demonstrated a defective autophagic pathway in sacsin-depleted cells [12]. Additionally, cellular and KO mouse models of ARSACS showed a reduced oxygen consumption rate (OCR), impaired mitochondrial fission and networking, and abnormal bundling of neurofilaments in many neuronal populations [8,10,11,39]. Similar disorganization of intermediate filaments has been observed in skin fibroblasts derived from ARSACS patients, and in KO HEK-293T and SH-SY5Y cells, all showing a collapsed perinuclear vimentin network [9,12]. In OCR studies, we observed impaired mitochondrial bioenergetics in *sacs^−/−^* compared with control larvae at the same stage of development, as evidenced by significant reductions in baseline respiration, ATP production, maximal respiration, proton leak, and spare respiratory capacity (Figure 3A). Furthermore, we showed a 25% increase in ROS production in *sacs^−/−^* embryos (Figure 3B,C), as well as a higher presence of apoptotic cells on acridine orange assay compared with the control group (Figure 3D,E). Taken together, these features suggest an increment of oxidative stress and activation of ROS-mediated apoptosis. Whilst the cross-species barrier prevented us from using human antibodies, qRT-PCR analysis in homozygous mutant zebrafish larvae at 120 hpf allowed us to observe a significant increase in *vim* and a significant reduction in *calr* mRNA expression compared with controls (Figure 3F,G), suggesting both potential cytoskeletal damage and putative impairments of cellular protein quality control and Ca^2+^ homeostasis. Notably, we have gathered preliminary proteomic data in KO SH-SY5Y cells that indicate reduced expression of human CALR (manuscript in preparation), a checkpoint in protein folding quality control. Finally, in line with previous data on a defective autophagic pathway in sacsin-depleted cells [12,40], we showed an upward trend of LC3 associated with significantly decreased p62 (* *p* ≤ 0.05) levels in *sacs* mutant larvae compared with control siblings (Figure 3H), also pointing to an impaired autophagic process [41,42]. Overall, these data are consistent with the features of ARSACS in *sacs^−/−^* mutant larvae and support the validity of the model as a platform for testing molecules with potential therapeutic effects.

### 2.4. Acetyl-DL-leucine and TUDCA Prevent Impairment of Locomotor Activity in Sacs^−/−^ Larvae and Enhance Responses to Light and Dark Transitions

**Figure 4:**
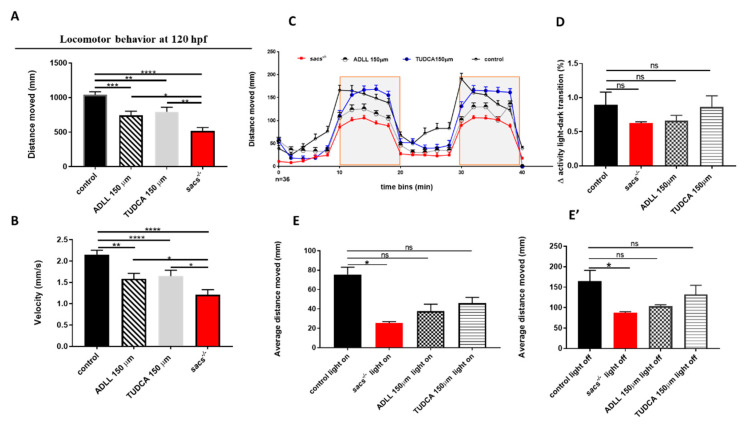
Treatment of homozygous *sacs^−/−^* mutants with TUDCA and ADLL is able to rescue the locomotor impairment seen in *sacs^−/−^* larvae. (**A**,**B**) Automated analysis of spontaneous motor activity of *sacs^−/−^* after drug treatments (untreated *sacs^−/−^ n* = 100; *sacs^−/−^* mutants treated with ADLL/Tanganil™ *n* = 150; *sacs^−/−^* mutants treated with TUDCA *n* = 150; controls *n* = 167) in 4 independent experiments. * *p* ≤ 0.05, ** *p* ≤ 0.01, *** *p* ≤ 0.001, **** *p* ≤ 0.0001were calculated by the Mann-Whitney test. (**C**) Swimming pattern of *sacs^−/−^* after drug treatments (untreated *sacs^−/−^ n* = 34; *sacs^−/−^* mutants treated with ADLL/Tanganil™ *n* = 34; *sacs^−/−^* mutants treated with TUDCA *n* = 34; controls *n* = 34). Each point in the graph represents the mean ± standard error of the mean of the distance moved by zebrafish larvae in 2-min time bins. The shaded parts represent the dark and the unshaded parts the light periods. The total number of embryos used for each group tested was 36. (**D**) Between-group differences in the average total activity were evaluated by comparing 1 min after and 1 min before light-to-dark and dark-to-light transitions. Values are represented as means ± standard error of the means. (**E**,**E’**) The average of the total activity of each group was measured during light on (**E**) and light off (**E’**) conditions. * *p* ≤ 0.05, calculated by Dunnett’s multiple comparisons test. Abbreviations: *n*, number of evaluated embryos in total; ns, not significant.

In the absence of FDA-approved medications for the treatment of degenerative ataxia [43], there are few options available to counteract the progressive degeneration of PC functions seen in ARSACS and similar clinical conditions [44]. Repurposing of FDA-approved drugs likely acting on multiple molecular targets seems a good avenue to explore at a preclinical level. In a pilot trial, we explored the feasibility of using *sacs^−/−^* larvae in drug screening, focusing on two potential targets. It has been speculated that stimulation of intracellular glutamate metabolism by branched-chain amino acids may improve neurotransmission among cerebellar neurons [45] and that acetyl-DL-leucine (ADLL, Tanganil™), a branched-chain amino acid used to treat the symptoms of acute vertigo, might have a positive effect on ataxic symptoms in cerebellar disorders, moreover with a low risk/benefit ratio, given the electrophysiological similarities and close interactions between vestibular and cerebellar neurons [46]. Interestingly, in open-label studies in other forms of inherited ataxia, ADLL has already been suggested to be efficacious [47], and it is also the subject of an on-going randomized controlled phase III trial [48]; however, it has never been tested in ARSACS. Similarly, TUDCA, a bile acid derivative with demonstrated beneficial neuroprotective effects in mouse models of neurodegeneration, including Parkinson’s disease [49], Huntington’s disease, and amyotrophic lateral sclerosis [50], appears to be of benefit in inherited ataxias [51]. TUDCA likely acts through attenuation of endoplasmic reticulum (ER) stress-induced apoptosis, and by counteracting the unfolded protein response and it is now entering a phase III clinical trial as an add-on therapy for ALS. Furthermore, it is worth noting that treatment with TUDCA also exerted a phenotype reversal in worm models of MJD/SCA3 (personal communication to FMS), a common form of dominant ataxia, and ameliorates proinflammatory polarization of microglia in vitro in multiple sclerosis in a dose-dependent manner [52]. TUDCA, too, has not been tested in ARSACS (either in patients or in mouse models). In view of this background, we investigated the potential of ADLL/Tanganil™ and TUDCA to counteract the phenotypes observed in *sacs^−/−^* larvae. For each substance, 4 hpf embryos through to 120 hpf larvae were exposed to five different concentrations in the range of 0.5 to 150 µM. We calculated the percentage of mortality at 30 hpf and assessed morphological alterations. Thereafter, the highest dose (150 µM) of each compound was used in the following experiments. We treated WT larvae at 120 hpf with both drugs and performed video tracking to measure the distance moved and velocity. The analysis did not show significant alteration or change in locomotor activity in each group of control larvae treated (Figure S2A), confirming the safety dose of these compounds. Then, we observed that 150 µM of ADLL/Tanganil™ or TUDCA could rescue, at least in part, the locomotor impairment seen in 120 hpf *sacs^−/−^* mutants, improving motor activity by 30% (Figure 4A,B). In addition, to evaluate the neuroactive properties of the substances tested, we exposed larvae at 120 hpf to an alternating light-dark locomotion test. This revealed changes in locomotor activity (Figure 4C,D) as described by others [53]. Although the data were not statistically significant, *sacs^−/−^-*treated larvae showed, on average, a tendency to an improvement in locomotor activity throughout the light/dark period (Figure 4E,E’).

### 2.5. ADLL and TUDCA Restore the Gene Expression Profile and Prevent Apoptosis in Sacs^−/−^ Larvae

**Figure 5:**
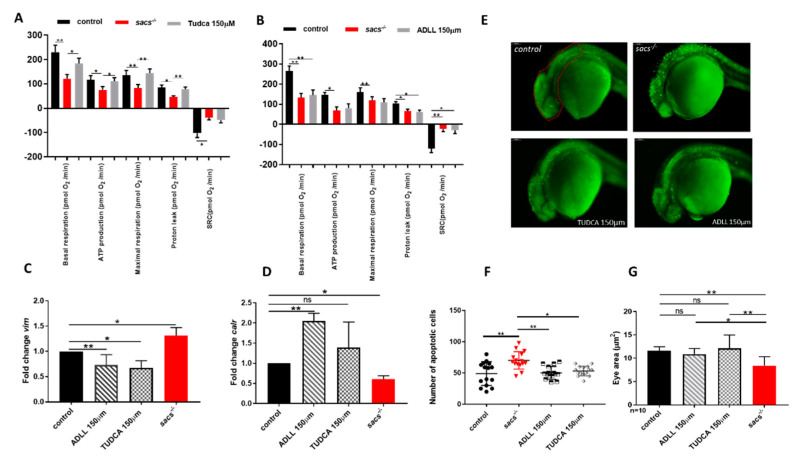
ADLL and TUDCA restore gene expression profile and prevent apoptosis in *sacs^−/−^* larvae. (**A**,**B**) Mitochondrial respiratory analysis after drug treatments. For each experiment, we compared treated *sacs^−/−^* larvae (*n* = 18) and untreated *sacs^−/−^* larvae (*n* = 18) and control (*n* = 18) at 120 hpf. * *p* ≤ 0.05, ** *p* ≤ 0.01, calculated by the non-parametric Kruskal-Wallis test. (**C**,**D**) qRT-PCR analysis on *sacs^−/−^* larvae after drug treatments. Three independent RNA samples from each group (controls, untreated *sacs^−/−^* and treated *sacs^−/−^*) were evaluated. * *p* ≤ 0.05, ** *p* ≤ 0.01, calculated by Dunnett’s multiple comparisons test. Abbreviation: ns, not significant (**E**) Detection of dying cells by acridine orange staining of 24 hpf *sacs^−/−^* embryos after drug treatments. The total number of embryos used per group tested (controls, untreated *sacs^−/−^* and treated *sacs^−/−^)* was 15. (**F**) Apoptotic cells were counted in the area defined by the red circle. Quantitative analysis of apoptotic cells. * *p* ≤ 0.05; ** *p* ≤ 0.01, calculated by the non-parametric Kruskal-Wallis test. The values are expressed as mean ± standard deviation. (**G**) Evaluation of the eye area of *sacs^−/−^* after drug treatments (controls, untreated *sacs^−/−^* and treated *sacs^−/−^*). * *p* ≤ 0.05, ** *p* ≤ 0.01, calculated by the non-parametric Kruskal-Wallis test. The values are expressed as mean ± standard deviation. Abbreviations: *n*, total number of evaluated embryos; error bars indicate standard errors of the means; ns, not significant.

To further validate the efficacy of ADLL/Tanganil™ and TUDCA in improving the phenotypes observed in sacs-null mutant zebrafish larvae, we tested OCR parameters, as well as vim and calr mRNA levels in controls, untreated *sacs^−/−^,* and treated *sacs^−/−^* larvae at 120 hpf. Our findings showed a partial correction of the bioenergetic deficit (Figure 5A,B) and the pattern of expression for both genes in treated *sacs^−/−^* larvae compared with untreated siblings (Figure 5C,D). The specificity of the drug’s activity in improving the phenotype in the *sacs^−/−^* model was further corroborated by trialing both ADLL/Tanganil™ and TUDCA in *sapje* larvae [54], a different model of motor impairment due to impaired dystrophin. In that model, no impact was observed either on mRNA levels or on motor performance (Supplementary Materials Figure S2). Finally, the pharmacological treatment of *sacs^−/−^* mutant embryos with ADLL/Tanganil™ and TUDCA reduced the number of apoptotic cells as shown by acridine orange staining at 24 hpf (Figure 5E,F) and appeared to increase the size of the eyes in treated mutant larvae (Figure 5G).

## 3. Discussion

ARSACS is considered the second most common form of autosomal recessively-inherited hereditary ataxia in Europe and Canada [55], and it remains an incurable condition, affecting a significant number of patients worldwide. Studies on the role of sacsin in patients and in disease models, and more generally, on its function in neurodegeneration, remain challenging but might represent a prerequisite to the design of preclinical treatments and pilot clinical trials in well-stratified groups of patients. Growing evidence supports the potential of the zebrafish as an efficient model for drug discovery, translational neuroscience, and disease modeling [14]. We employed CRISPR/Cas9-mediated gene editing to knock out the zebrafish *sacs* gene. *SACS* is a gene highly conserved throughout evolution in vertebrates, as supported by evidence of similar expression patterns in mammals [1,4]. In zebrafish, sacsin shows 35% identity with human protein [7]. We previously showed that in 24 hpf embryos, *sacs* mRNA localized at the level of somites, in the head mesoderm/neural crest, and in the midbrain regions, suggesting a higher expression in metabolically active tissues, as also demonstrated by qRT-PCR analysis [7]. The duplicated zebrafish *sacs2* transcript (ID ENSDART00000162761.2) has a low degree of synteny with the human one. Therefore, in the current research, we established and characterized a stable *sacs*-null mutant zebrafish model. From post-mortem studies in ARSACS and KO mice, it appears clear that loss of sacsin results in pathological changes in the cerebellum [29] associated with significant depletion of PCs, which together cause motor defects and muscle atrophy [10]. The overall architecture and cell types of the cerebellum are highly conserved from teleost fish to mammals [56,57]. In the zebrafish, the hindbrain develops, differentiates, and reaches functional maturity through embryonic and larval stages, during which these fish are almost transparent; this allows whole-brain analysis and makes them a useful tool for in vivo bioimaging [57,58]. PCs are the main neuronal population and the sole output of the cerebellum in zebrafish [34]. We generated our *sacs* mutant zebrafish model in the genetic background of a PC reporter strain of zebrafish, to exploit the absence of sacsin effects on PCs and the cerebellum. The *sacs-*null larvae appeared morphologically normal but showed a slightly different phenotype, in that the eye appeared smaller than in controls. Consistent with what has been observed in other models, the “cerebellum” of *sacs^−/−^* larvae appeared smaller and thinner compared with that of control larvae at 120 hpf, probably due to loss of PCs, which in our mutant was related to the impairment of Ca^2+^ signaling in these cells, a potential key element leading to cerebellar dysfunction. Real-time monitoring of intracellular Ca^2+^ dynamics in *sacs^−/−^* PCs raised in the Tg(tagRFP-T:PC:GCaMP5G) background allowed us to detect an increase in Ca^2+^ fluorescence fluctuations. An intracellular Ca^2+^ increase is associated with necrosis, responsible for a derangement of cell integrity and function, and excitotoxicity, a state in which glutamate-dependent hyperstimulation leads neurons to necrotic death [59]. Our findings support the idea that PC dysfunction impairs early motor function in a manner reminiscent of what occurs in ARSACS [60]. Muscle weakness and deficits in locomotor activity are prominent symptoms in affected children, and mouse models, too, manifest motor defects and muscle weakness [11]. Our mutant larvae showed strongly reduced motor activity at 120 hpf, mimicking the motor deficit seen in murine models. Contrary to *Sacs*-KO mice, which, from 120 days of age, exhibit progressive muscle weakness compared with age-matched controls [10,11], in zebrafish, we did not observe any muscular degeneration (Supplementary Materials Figure S1) or abnormalities in axon branching, or any reduction in axon length on syt2 immunostaining. Moreover, acetylated α-tubulin antibody staining, employed to analyze sensory neuron development, showed no abnormalities in *sacs*^−/−^ mutants [61]. The analyses in zebrafish were carried out much earlier in their development (larval stage) than in mice and we therefore cannot exclude the possibility of a more severe motor neuron phenotype appearing in adult fish.

The sequence of events that leads to neuronal cell death in ARSACS is still unclear, and several studies have pointed out that cytoskeletal disorganization could be an early phenomenon [10]. Girard and colleagues provided evidence that sacsin localizes to mitochondria, and that targeted disruption of this protein causes alterations in mitochondrial morphology and function [8]. It has been demonstrated that the absence of sacsin observed in human dermal fibroblasts from ARSACS patients causes abnormal vimentin (a principal constituent of the intermediate filament family of proteins) bundles accompanied by morphological alterations in mitochondrial networks [9]. Gene expression analysis revealed increased *vim* mRNA expression in *sacs^−/−^* mutant zebrafish larvae compared with controls. Dysfunctional mitochondrial dynamics in ARSACS patients is also associated with impairment of micro-oxygraphy parameters and increased oxidative stress [62]. Combined with impaired expression of the ER-related protein calreticulin, and changes in markers of the autophagic process, the bioenergetic defects seen in the null larvae further validate the teleost as a new model of the human disease. Calreticulin is a chaperone playing multiple roles in several cell processes, such as protein folding quality control and Ca^2+^ homeostasis, and it is also an ER stress response indicator [63]. Salati and colleagues suggested that the absence of calreticulin increased oxidative DNA damage [64]. In vivo detection of ROS on *sacs^−/−^* larvae revealed higher ROS production in the mutant larvae. Besides disrupting mitochondrial membrane integrity, membrane potential, and the respiratory chain, ROS accumulation activates apoptosis in a caspase-dependent manner [65]. Whilst apoptosis occurs naturally during nervous system development [66], we observed that *sacs-*null mutant embryos displayed an increase in apoptotic cells, which were found to be densely distributed mainly in the eye area at 48 hpf, a finding likely associated with the slight “microphthalmia” observed in mutants [66]. Altogether, the findings seen in zebrafish lend further support to the hypothesis that absence of sacsin leads to defects in mitochondrial trafficking, and thus to accumulation of aberrant mitochondria in PCs, which, it is suggested, disrupts Ca^2+^ homeostasis probably in distal dendrites of PCs, stimulating ROS buildup and inducing autophagy processes to remove damaged mitochondria. The zebrafish model we generated could help to further fine tune the chain of events associated with sacsin depletion in the early and late stages of the disease and offer further insights into the role of sacsin during brain development.

Given the availability of several cell models and two murine strains in which sacsin is deficient, the need for a further tool might be questioned. However, the new model herein described, by replicating the phenotype of ARSACS, addresses the urgent need to find systematic approaches to facilitate drug discovery. Having the advantage of allowing rapid assessment of morphological and behavioral readouts in live animals, the zebrafish is indeed pivotal for in vivo high-throughput pharmacological screening [67]. Because of their relatively lower costs and easy handling, zebrafish could be used for prioritization of drugs and compounds before moving on to more expensive murine studies. To assess this, we performed proof-of-principle studies investigating the potential role of ADLL/Tanganil™ and TUDCA. ADLL modulates glutamate neurotransmission in the cerebellum through the branched-chain amino acid transferase, which is important both for glutamate release during excitation and for the activation of metabotropic glutamate receptors required for cerebellar plasticity [68]. TUDCA has neuroprotective effects [69], acting as a mitochondrial stabilizer and antiapoptotic agent in several models of neurodegeneration [70]. It is able to cross the blood–brain barrier in humans [71], and is now entering a phase III clinical trial as an add-on therapy for ALS (ClinicalTrials.gov Identifier: NCT03800524). Our treatments in *sacs^−/−^* fish recorded increased swim distance and velocity, partial restoration of *vim* and *calr* mRNA expression levels, improved SRC and basal ATP levels, as well as a significant reduction in apoptotic cells. These improvements appeared specific to our model associated with reduced PC size as they did not occur in a different zebrafish strain (*sapje*) presenting altered locomotor behavior due to muscle damage [54]. Hence, even though these pilot drug treatments did not fully complement or “cure” the phenotypes (locomotor or molecular) of mutant larvae, they demonstrated the potentialities of the system for future high-throughput screening studies. In summary, this paper describes the generation of a stable zebrafish *sacs*-null line, a further model of sacsinopathy. Although “humanizing” fish data is always tricky, we obtained findings replicating the main “clinical” and biological features seen in children with ARSACS. However, a potential weakness of our study might arise from the differences in cerebellar cytoarchitecture between zebrafish and mammals [24]. Even considering this limitation, these zebrafish larvae, expressing a fluorescent reporter and offering the possibility of real-time monitoring of intracellular Ca^2+^ dynamics, provide a tool for in vivo analysis at high subcellular and temporal resolution in the native context of the cerebellar circuitry. In addition, contrary to *Sacs*-null mice [10], *sacs^−/−^* larvae proved useful for investigating earlier steps in locomotor impairment and cerebellar area reduction. We believe that *sacs-*deficient zebrafish embryos might facilitate study of the consequences of sacsin disruption on neurodevelopment. Even though further research in adult fish will be necessary, *sacs^−/−^* larvae will likely help to pave the way for drug discovery studies in vivo and allow preliminary testing of new treatment paths before embarking on more costly studies in mice [72].

## 4. Materials and Methods

### 4.1. Zebrafish Husbandry

Experiments were carried out using transgenic lines of the Tg(tagRFP-T:PC:GCaMP5G) strain, kindly provided by Prof. Reinhard Köster (University of Braunschweig, Germany) [24], and the *sapje* mutant strain (a validated model of Duchenne muscular dystrophy), kindly provided by the laboratory of Prof. Simon Hughes (King’s College, London, UK) [54]; we also used the wild-type AB strain. Adults were housed in tanks at a density of no more than five zebrafish per liter at a constant temperature of 28 °C on a 14-h light/10-h dark cycle. Zebrafish eggs and embryos were collected and raised at 28.5 °C in E3 medium using established procedures and staged in hours post fertilization (hpf) or days post fertilization (dpf) [73]. The generation of the CRISPR/cas9 mutant was carried out under the ethical approval n° 338/2020-PR of the Italian Minister of Health, in accordance with the European Union (EU) Directive 2010/63/EU on the protection of animals used for scientific purposes, and under the supervision of the Institutional Animal Care and Use Committee of the University of Pisa, and complied with the 3R principles [18].

### 4.2. Multiple Alignments of Sacsin Amino Acid Sequences

Multiple alignments of sacsin amino acid sequences were performed using Clustal Omega (https://www.ebi.ac.uk/Tools/msa/clustalo/, October 2018) for the following organisms: *Danio rerio* sacs (ENSDARG00000091042.4); *Danio rerio* si:dkeyp-118h9.7 (ENSDART00000162761.2); *Homo sapiens* sacsin (ENSG00000151835.16) (HADY01011608.1); and *Mus musculus* sacsin (ENSMUSG00000048279.19).

### 4.3. Establishing the Mutant Line

The selected sgRNA was chosen among the top targets identified by CHOPCHOP software (www.chochop.rc.fas.harvard.edu/index.php, November 2018) set with NGG PAM sites and zero predicted off-targets (fewer than three mismatches in the *sacs*-targeting 20-mer). The sgRNA was designed against exon 7 of the *sacs* transcript (ENSDARG00000091042.4) and generated as already described [74]. The sgRNA was transcribed using the Megascript T7 Transcription kit (Invitrogen, Heidelberg, Germany). The optimized Cas9 mRNA, for genome editing in zebrafish, was transcribed from linearized template plasmid pCS2-nCas9n using the mMESSAGE mMACHINE™ SP6 Transcription kit (ThermoFisher Scientific, Waltham, MA). RNA concentration was quantified using a NanoDrop spectrophotometer (Optosky, Xiamen, China) and diluted to 500 ng/µL. About 100 ng of *sacs*-sgRNA and 500 ng of Cas9 mRNA were co-injected into 1-cell stage embryos, to ensure high-efficiency delivery of the injected mRNA to the embryo. The injected volume was ~1 nL of solution. At least three independent injection experiments were performed with spawns from different founder fish to control for batch effect.

### 4.4. Genotyping

For mutation screening, sgRNA-injected F0 embryos were raised to adulthood and outcrossed with Tg(tagRFP-T:PC:GCaMP5G) adults to obtain F1 heterozygous embryos. To identify potential adults carrying mutations, PCR and fragment analysis using genomic DNA from 16 randomly selected F1 embryos were performed using the following primers: *Forward* 5′-TTTGTTTTCTCCCTTTGCCACTT-3′; *Reverse* 5′- GATCAGGCCAGGCTCCATAAATA-3′. F1 heterozygous fish carrying a 10-bp deletion mutation in the targeted site were selected and inter-crossed to generate the F2 homozygous *sacs^−/−^* line.

### 4.5. Quantitative Reverse Transcription Polymerase Chain Reaction (qRT-PCR)

Total RNA was extracted from 30 embryos at 120 hpf using the Quick RNA Miniprep kit (Zymo Research, Irvine, CA) according to the manufacturer’s instructions. cDNA and qRT-PCR were performed as described elsewhere [75]. Relative expression levels of each gene were calculated using the 2^-ΔΔCt^ method [76]. The results obtained in at least three independent experiments were normalized to the expression of the housekeeping gene, *β-actin* (ENSDARG00000037746). The mean of the controls was set at one.

### 4.6. Immunohistochemistry Staining of Whole-Mount Zebrafish Embryos

To prevent the development of pigmentation, embryos were treated with 0.005% phenylthiourea from 24 hpf. Whole-mount immunohistochemistry was performed in 48 or 120 hpf embryos fixed in 4% PFA overnight at 4 °C and stored in methanol as described in [56]. The antibodies used were mouse anti-Znp1 (ab113545, Abcam, Cambridge, MA,1:200 dilution), mouse anti-acetylated-tubulin (018M4788V, Life Technology, Monza, IT, 1:500 dilution), and mouse anti-Parvalbumin 7 (a kind gift from Hibi Lab, Nagoya University, JP, 1:200 dilution) [77].

### 4.7. Analysis of Larval Morphology

Live zebrafish were mounted on glass depression slides with 3% agarose. Images were obtained using a Leica M205FA stereomicroscope (Leica Microsystem, WetzlarGermany). The body length and eye size of 120 hpf larvae were measured using ImageJ 64 software [78]. Images of the hindbrain area were acquired using the Tg(tagRFP-T:PC:GCaMP5G) stable transgenic line, and the area in µm^2^ of the region of interest (ROI) was calculated using ImageJ 64 software.

### 4.8. Locomotor Behavior

Coiling behavior was measured in 30 hpf embryos using Danioscope software (Noldus^©^, the Netherlands). We also analyzed locomotion in 120 hpf larvae in each experimental group. The larvae were transferred into 96-well plates containing 300 μL of egg water per well. Each plate was placed in the DanioVision^®^ device (Noldus^©^ Information Technologies, Wageningen, The Netherlands) and the larval activity was recorded for 30 min and analyzed using EthoVision XT^®^ software (Noldus^©^ Information Technologies, Wageningen, The Netherlands) [79]. Statistical analysis was performed considering five independent biological replicate experiments and the data were plotted as the mean ± standard error of the mean.

### 4.9. Mitochondrial Respiratory Analysis

Mitochondrial respiration was analyzed in untreated and treated homozygous *sacs^−/−^* larvae at 120 hpf using the XF24 extracellular flux analyzer (Seahorse Bioscience, North Billerica, MA.). The dual analyte sensor cartridges were soaked in XF calibrator solution (Seahorse Bioscience) in 24-cell culture overnight at 28 °C to hydrate. About 30 min before the trial period, the appropriate injection cartridges were reloaded. The following chemicals were used for this experiment: oligomycin at a concentration of 25 µM, FCCP at a concentration of 5 µM, and rotenone plus antimycin A at a concentration of 5 µM. The 120 hpf larvae were staged and placed in 20 of the 24 wells of an islet microplate. The islet plate acquisition screens were placed on the measurement area to hold the larvae in place. Four wells were left empty as a control. Each well was filled with 500 µL of egg water (pH 7.4). Basal respiration, ATP production, maximal respiration rate, and spare respiratory capacity were measured using a standard approach [33].

### 4.10. Calcium Imaging

Zebrafish larvae at 120 hpf were restrained in low melting point agar and a Nikon FN1 microscope (Nikon, Tokyo, JP) was used for video recording; the image acquisitions were obtained using a Prime sCMOS camera (Teledyne Photometrics, Tucson, AZ.)) supplied with Metafluor software (Molecular Devices, San Jose, CA), applying a time-lapse interval of 150 ms and acquiring 3860 frames per video. The distribution of fluorescence fluctuations (ΔF/F0) in the hindbrain area was evaluated in a pre-defined ROI using ImageJ 64 software. Data were normalized to background fluorescence and were quantified by the Pearson’s coefficient of skewness defined as: δ = (3(M–m))/σ, where M is the mean, m is the median, and σ is the standard deviation of the distribution as described elsewhere [37].

### 4.11. Oxidative Stress Measurement

Reactive oxygen species (ROS) levels were determined using an in vivo carboxy-H2DCFDA fluorescent probe (#8206004, Abcam, Cambridge, MA). Zebrafish embryos at 48 hpf were incubated with 30 µM of this probe for 40 min in the dark and then washed three times with E3 medium. A lateral image of each larva was acquired using a fluorescence microscope, and the fluorescence intensity in the selected ROI was quantified using ImageJ 64 software. Data were normalized to background fluorescence.

### 4.12. Western Blotting

Embryos collected at 48 hpf were dechorionated, deyolked, and lysed in radioimmunoprecipitation assay buffer (RIPA buffer) supplemented with 1 mM PMSF, 1 mM sodium fluoride, and 1 mM sodium vanadate (Na_3_VO_4_). Equal amounts of embryo protein (50 μg) were electrophoresed in 10% SDS-PAGE gel and transferred to nitrocellulose membranes. Western blotting was performed as previously described [80,81], and the primary antibodies used were: rabbit anti-β-tubulin (#2146, Cell Signaling Technology, Danvers, MA, 1:1000), rabbit anti-LC3 (L7543, Sigma-Aldrich, MI, IT, 1:1000), and rabbit anti-p62 (GTX100685, GeneTex, Irvine, CA, 1:500). The *p*-value was calculated using GraphPad Prism 6 software (San Diego, CA, USA).

### 4.13. Detection of Apoptotic Cells

In each group, apoptotic cells from larvae at 24 and 48 hpf were detected by staining with acridine orange (#235474, Sigma-Aldrich,**** St. Louis, MO ). Zebrafish embryos were incubated with 10 μg/mL acridine orange solution for 15 min in the dark; the larvae were then washed three times with E3 medium. At 48 hpf, we counted acridine orange-positive cells within a pre-defined area and a quantitative analysis was performed as described elsewhere [78,82].

### 4.14. Pharmacological Treatments

Normally developing embryos were selected under a stereomicroscope at 4 hpf and randomly placed in 60 mm × 15 mm petri dishes at a density of 50 per dish, each dish containing one of two specific drugs diluted in egg water. Stock solutions of tauroursodeoxycholic acid (TUDCA), sodium salt (#14605-22-2, Calbiochem, San Diego,), and N-acetyl-L-leucine (#MKCK6900, Sigma-Aldrich,**** St. Louis, MO) were prepared in Milli-Q water (Merck-Millipore, Milan, Italy) and diluted in egg water to the final administered concentrations. To select the appropriate working dilution for each substance, a preliminary dose-dependence test was performed using wild-type embryos. Four to five different concentrations in the range of 0.5 to 150 µM were tested for each substance. The highest concentration (150 µM) of each compound was chosen as the working dilution for all pharmacological experiments. To verify the efficacy of the molecules tested, we performed the aforementioned locomotor behavior assay. After 30 min of adaptation, larval locomotion was analyzed for 40 min, through 4 cycles of alternating light and dark periods [53,83]. For each experiment, at least three independent assays were performed.

### 4.15. Birefringence Assay

Muscle birefringence, linked to myofibril organization, was analyzed by placing anesthetized embryos on a glass polarizing filter, covering them with a second polarizing filter, and recording them under a Leica M205FA microscope as described elsewhere [84,85]. Embryos were photographed in a bright field.

### 4.16. Statistics

All data in the manuscript represent three or more independent experiments giving similar results. We performed the statistical analysis using GraphPad Prism 6 software. The significance between groups was determined using Dunnett’s multiple comparisons test or the non-parametric one-tailed Mann-Whitney rank sum test, as indicated in each figure legend. Statistical analysis for qRT-PCR experiments was performed using the two-tailed paired Student’s *t*-test. Statistical significance is reported as: * *p* ≤ 0.05, ** *p* ≤ 0.01, *** *p* ≤ 0.001, or **** *p* ≤ 0.0001.

## Supplementary Materials

The following are available online at https://www.mdpi.com/article/10.3390/ijms22168401/s1.
