# Supplementary material for: Efficient Neuroprotective Rescue of Sacsin-Related Disease Phenotypes in Zebrafish
Source: Int J Mol Sci. 2021 Aug 5;22(16):8401. doi: 10.3390/ijms22168401 (PMC8395086; doi:10.3390/ijms22168401)
Supplement: Supplementary file 1 [file ijms-22-08401-s001.zip › Supplementary material revision.pdf]

Supplementary information (Supplementary Figure S1, Supplementary Figure S2)

Figure S1. **A)** Percentages of similarity of amino acid sequences**. B)** Dorsal view photographs of representative control and *sacs*$^{-/-}$ larvae. No dysmorphology was noted at 120 hpf but homozygous larvae showed a slight reduction of the eye area. **(C)** Statistical analysis of the data shown in **(B)**. **D)** Lateral views of whole-mount embryos labeled with mAb Znp-1 (*sacs*$^{-/-}$ n=25; controls n=25). **E)** Lateral views of whole-mount embryos stained with acetylated tubulin mAb in *sacs*$^{-/-}$ and control embryos at 48 hpf (*sacs*$^{-/-}$ n=20; controls n=20). **F**) Muscle organization determined with birefringence in control and *sacs*$^{-/-}$ larvae at 120 hpf. Birefringence images were obtained by viewing zebrafish with a plane polarizing filter. **G)** Detection of dying cells by acridine orange staining in the tail region of control and *sacs*$^{-/-}$ mutant embryos at 48 hpf (lateral views). Quantitative analysis of apoptotic cells. Abbreviations: n, number of evaluated embryos in total; error bars indicate standard errors of the means (SEM): ns, not significant. The values are expressed as mean ± SEM. Statistical analysis was performed by Mann-Whitney test.

Figure S2. A-B) Automated analysis of spontaneous motor activity of sacs WT larvae at 120 hpf after drug treatments (untreated sacs WT n=40; sacs WT treated with ADLL/Tanganil™ n=40; sacs WT treated with TUDCA n=40) in 2 independent experiments. The values are expressed as mean ± SEM. Statistical analysis was performed by Mann-Whitney test. Abbreviations: ns, not significant.

C-D)RT-qPCR analysis on homozygous *sapje* zebrafish larvae and controls. Three independent RNA samples from each group were evaluated at 120 hpf. The mRNA expression levels of *vim* and *calr* were normalized to *β-actin*. **E-F)** Automated analysis of spontaneous motor activity of *sapje* after drugs treatment (untreated *sapje* n=29; *sapje* treated with ADLL/Tanganil™ n=20; *sapje* treated with TUDCA n=20). ns, not significant. The values are expressed as mean ± SEM. Statistical analysis was performed by Mann-Whitney test. Abbreviations: ns, not significant.

Figure S3. Full-length lanes of original blots referring to Figure3H. Dotted lines indicate molecular weight

corresponding to the cut on the PVDF-membrane. Loaded samples not related to this work were removed and not shown.

Supplementary Video S1: Representative video of sacs$^{-/-}$ mutant zebrafish larvae at 120 hpf. Line A-D (sacs WT larvae); Line E-H (sacs$^{-/-}$ larvae).
