# Supplementary figures and images for: Efficient Neuroprotective Rescue of Sacsin-Related Disease Phenotypes in Zebrafish
Source: Int J Mol Sci. 2021 Aug 5;22(16):8401. doi: 10.3390/ijms22168401 (PMC8395086; doi:10.3390/ijms22168401)

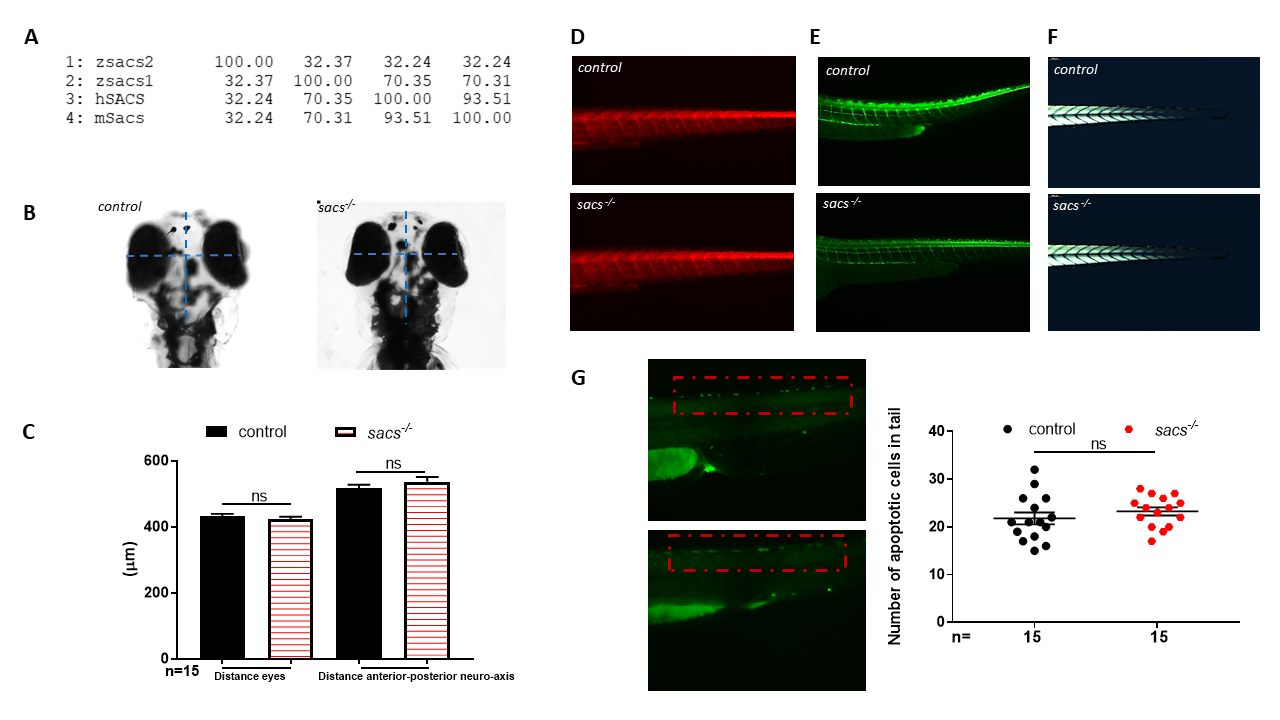

Supplement: Supplementary file 1 [file ijms-22-08401-s001.zip › Supplem Fig.S1.tif]

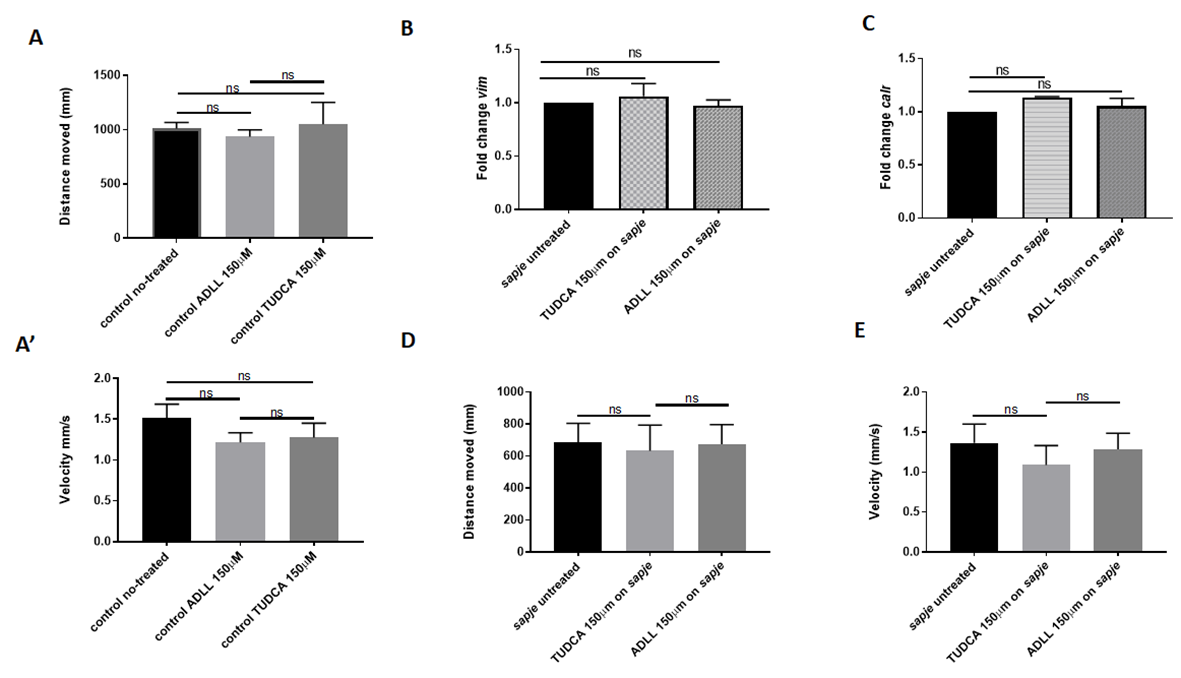

Supplement: Supplementary file 1 [file ijms-22-08401-s001.zip › Supplem Fig.S2.tif]

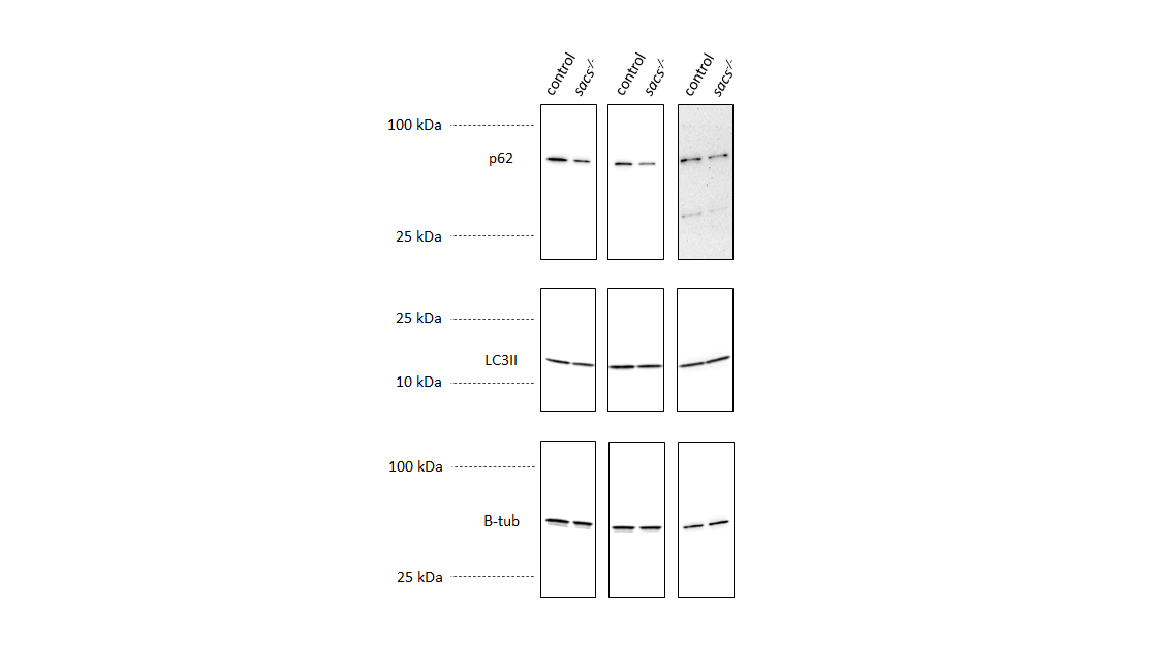

Supplement: Supplementary file 1 [file ijms-22-08401-s001.zip › Supplem. Fig.S3.tif]

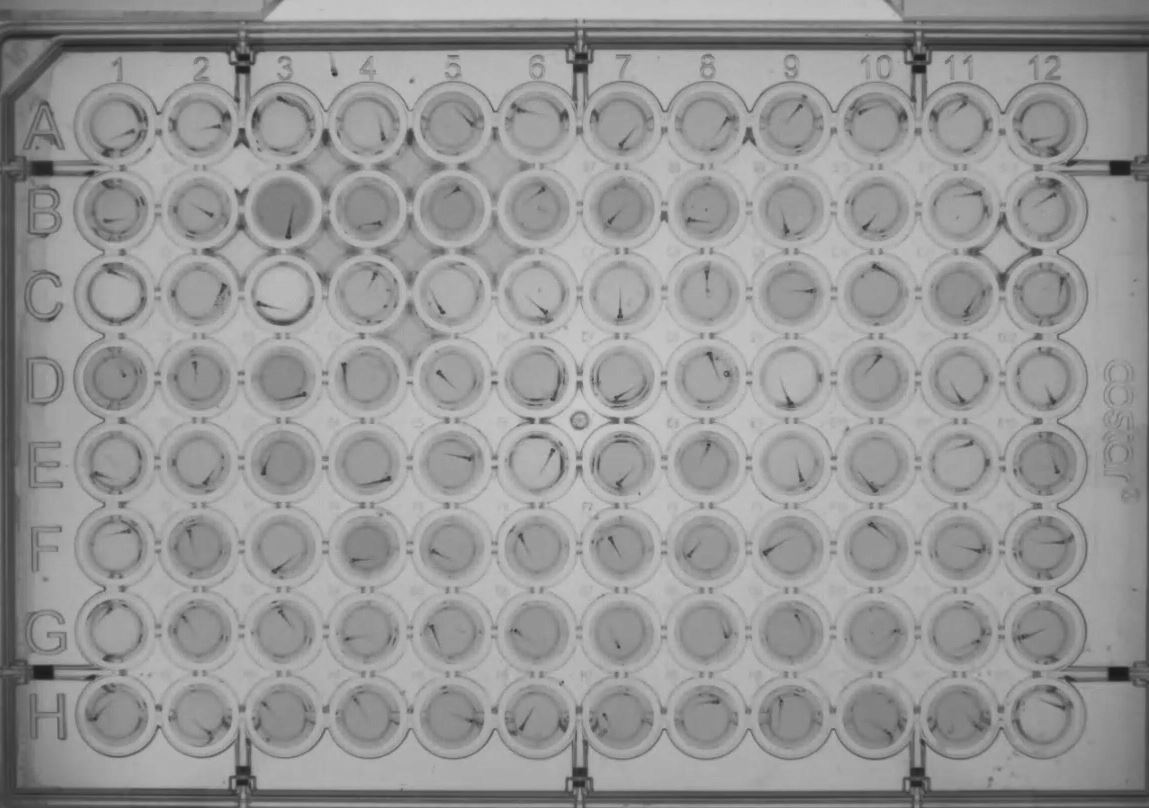

Supplement: Supplementary file 1 [file ijms-22-08401-s001.zip › Supplementary Video Snapshot of tracking.JPG]
